# Quantitative proteomic analysis reveals potential serum diagnostic markers for colorectal adenoma

**DOI:** 10.3389/fmolb.2025.1628587

**Published:** 2025-11-24

**Authors:** Chengli Yu, Xin Huang, Yating Cao, Qiuxia Yang, Ting Liu, Jingjie Zhou, Juping You, Ye Zhang, Ailing Yin, Haibing Hua

**Affiliations:** 1 Jiangsu Key Laboratory for Functional Substances of Chinese Medicine, School of Pharmacy, The Affiliated Jiangyin Hospital of Nanjing University of Chinese Medicine, Nanjing University of Chinese Medicine, Nanjing, China; 2 Nanjing Hospital of Chinese Medicine Affiliated to Nanjing University of Chinese Medicine, Nanjing, China; 3 The Affiliated Jiangyin Hospital of Nanjing University of Chinese Medicine, Jiangyin, China; 4 State Key Laboratory of Natural Medicines, China Pharmaceutical University, Nanjing, China

**Keywords:** colorectal adenomas, inflammatory polyps, serum, biomarker, FLNA

## Abstract

**Introduction:**

Colorectal cancer (CRC) is one of the leading causes of cancer-related death, and most CRCs arise from colorectal adenomas. Early detection and removal of precancerous lesions during the adenoma-carcinoma sequence can significantly reduce CRC risk. However, current clinical practice lacks rapid, noninvasive screening tools for reliable adenoma detection.

**Methods:**

Proteomic analysis was performed on serum samples from patients with inflammatory polyps (non-neoplastic), patients with adenomas, and healthy controls to identify key differentially expressed proteins capable of distinguishing adenoma patients. The alterations in these candidate proteins were further validated by ELISA to evaluate their potential as diagnostic biomarkers for colorectal adenoma.

**Results:**

In two independent cohorts, we identified two candidate biomarkers, apolipoprotein A4 (APOA4) and filamin A (FLNA), through a multi-step selection process involving ANOVA p-value screening, sparse partial least squares discriminant analysis (sPLS-DA), and LASSO regression analysis. These candidates were subsequently validated in a third cohort using ELISA. The ELISA results for APOA4 were discordant with the liquid chromatography-tandem mass spectrometry (LC-MS/MS) findings. In contrast, FLNA levels measured by ELISA showed a progressive decrease from healthy controls to patients with inflammatory polyps and further to those with adenomas. We propose FLNA as a potential biomarker for the diagnosis of colorectal adenomas. The areas under the ROC curves exceeded 0.7 for both key clinical comparisons: 0.810 for adenomas versus healthy controls, and 0.734 for adenomas versus inflammatory polyps.

**Discussion:**

Overall, this study not only enhances our understanding of the serum proteome in colorectal adenoma but also identifies FLNA as a promising biomarker for its clinical diagnosis.

## Introduction

Colorectal cancer (CRC) represents the third most prevalent malignancy globally and ranks as the second leading cause of cancer-related mortality (Bray et al., 2024). The majority of CRCs arise from colorectal polyps - abnormal epithelial proliferations forming protrusions from the mucosal surface (Abu Bakar et al., 2024). Colorectal polyps are categorized as either non-neoplastic or neoplastic. Non-neoplastic polyps comprise hyperplastic polyps, inflammatory polyps, juvenile polyps, and hamartomatous polyps. Neoplastic polyps primarily refer to adenomas (Rubio et al., 2002). Notably, epidemiological evidence indicates that only about 5% of colorectal polyps progress to CRC and about 85% of CRC cases originate from adenomatous polyps (Strum, 2016), which are well-established precancerous lesions demonstrating malignant transformation potential. This adenoma-carcinoma sequence underscores the critical importance of adenoma detection through screening programs as an effective strategy for CRC prevention.

Colonoscopy remains the gold standard for colorectal polyp diagnosis and treatment, offering direct visualization of the intestinal lumen while enabling simultaneous biopsy and therapeutic intervention. This procedure plays a pivotal role in both the diagnosis of colorectal polyps and the prevention of colorectal cancer (CRC). Compelling evidence has established that a higher adenoma detection rate is associated with a reduced risk of post-colonoscopy colorectal cancer (Schottinger et al., 2022). However, its clinical application is constrained by several limitations, such as accessibility, quality of bowel preparation, endoscopist experience, and polyp size and location. These constraints contribute to delayed patient management and missed diagnoses, highlighting the need for complementary, less invasive screening approaches. The development of more accessible testing methods could significantly expand screening coverage, improve adenoma detection rates, and ultimately reduce the burden of colorectal cancer through early intervention.

Serum can be obtained through non-invasive methods, and offers distinct advantages including safety, low cost, and technical feasibility. This biofluid contains a rich repertoire of proteins and peptides originating from cellular secretion, tissue leakage and proteolytic processing. These molecular constituents reflect the host’s physiological and pathological status. Currently, highly sensitive and specific biomarkers for differentiating colorectal adenomas from both healthy individuals and non-neoplastic colorectal polyps remain unavailable. Therefore, the data-independent acquisition (DIA) mass spectrometry method was used for quantitative comparison of serum proteomes across inflammatory polyp patients, adenoma patients and healthy controls, to identify clinically applicable serum biomarkers for colorectal adenoma screening and complementary diagnosis.

## Methods

### Human serum samples

Serum samples of healthy individuals and patients with inflammatory colorectal polyps and colorectal adenomas were collected from the Affiliated Jiangyin Hospital of Nanjing University of Chinese Medicine. The physicians requested the medically indicated blood collection. All participants were diagnosed using colonoscopy, and individuals with cancer, cardiovascular diseases, immunodeficiency diseases, and other nervous system diseases that may impact metabolism were excluded. Patients with a history of long-term drug use were also excluded. Patients and healthy subjects gave written informed consent before enrollment. All procedures were approved by the medical ethics committee of the Affiliated Jiangyin Hospital of Nanjing University of Chinese Medicine in accordance with the Declaration of Helsinki. Prior to colonoscopy, blood samples were obtained from all patients. All blood samples were promptly centrifuged at 2,000 × g for 15 min, and serum was aliquoted into clean Eppendorf tubes and kept at −80 °C before analysis.

### Sample preparation for proteomic analysis

To minimize the inter-individual variation, we performed sample pooling before processing. Three to six individual samples from the same group were pooled to constitute a pool. The sequential precipitation and delipidation method was used for the depletion of high-abundance proteins (Li et al., 2020). A total of 50 μL of water and 250 μL of methyl tert-butyl ether (MTBE) (Sigma, 306975) were added to 50 μL of the pooled serum, followed by vortex mixing. Subsequently, 150 μL of methanol (Merck, 1.06007.4008) was added and the mixture was incubated at 4 °C for 30 min. After incubation, the mixture was centrifuged at 21,000 × g for 30 min at 4 °C, and the supernatant was carefully transferred to a new tube. Then 500 μL of MTBE and 100 μL of water were added. After efficient mixing, the solution separated into two phases. The lower phase was collected and desalted using an HLB SPE column (Waters, 186000383) according to the manufacturer’s instructions.

The resulting samples were resuspended in 8 M urea (Sigma, U5128)/100 mM NH_4_HCO_3_ (Honeywell, 40867) and reduced with 20 mM dithiothreitol at 37 °C for 1 h followed by alkylation with 40 mM iodoacetamide for 30 min at room temperature in the dark. The urea concentration was diluted to less than 2 M with four volume of 50 mM NH_4_HCO_3_. The trypsin was added at a trypsin:protein ratio of 1:50 (w/w) for digestion at 37 °C overnight. Samples were acidified with trifluoroacetic acid to a final concentration of 1% for C18 StageTip binding and desalting. The eluted peptides were lyophilized and subsequently reconstituted in 0.1% (v/v) formic acid solution. Following this, equal sample aliquots were subjected to LC-MS/MS analysis.

### LC-MS/MS analysis

All samples were analyzed in data independent acquisition mode, and 2 μg peptides were loaded on 20-cm column packed in-house with C18 3 um ReproSil particles (Dr. Maisch GmbH, r13.aq.). Peptide mixtures were injected on an EASY-nLC 1200 system (Thermo Fisher Scientific) coupled to the mass spectrometer (Q Exactive Plus, Thermo Fisher Scientific), and separated by a non-linear 120 min gradient using mobile phase A (100% H2O, 0.1% formic acid) and B (80% acetonitrile, 0.1% formic acid). Peptides were eluted (300 nL/min) by gradient as follows: 2%–5% B, 5 min; 5%–32% B, 90 min; 32%–45%, 12 min; 45%–100% B, 3 min; 100% B, 10 min. Column temperature was maintained at 50 °C. The survey scan changes in the 300–1,500 m/z range with a maximum injection time of 150 ms and a resolution of 70,000. The automated gain control (AGC) target was 3e6. Following the full MS scan, DIA scans were acquired at a resolution of 35,000 and AGC target 2e5 with 20 m/z isolation window. The precursors were fragmented using HCD and normalized collision energy set to 27%, and maximum injection time was automatic. The data were recorded in centroid mode.

### Data analysis

Raw files were analyzed with DIA-NN (version 1.8.1). The spectral library was created using the human proteome downloaded from Uniprot (retrieved in March 2023) with the ‘Deep learning-based spectra and RTs prediction’ enabled. The false discovery rate (FDR) of precursor was set as 1%. Other settings were used as default parameters. Statistical analyses and graphical representations were performed in R (https://www.r-project.org/). All bioinformatics analyses were done with R (https://www.r-project.org/), Origin and Metascape (Zhou et al., 2019). In each experimental group, more than half of the proteins with quantitative values are retained. Missing values are imputed using the KNN algorithm. Batch effects were removed with R package sva from the proteins quantified across both cohort 1 and cohort 2. Principal component analysis (PCA) was performed using the R package factoextra, and results were visualized in Origin. We performed sPLS-DA (KA et al., 2011) and LASSO regression, employing the R packages mixOmics and glmnet, respectively. Receiver operating characteristic (ROC) curve analysis was carried out with the pROC package. Protein abundance was compared using ANOVA test with 5% FDR. P values were adjusted by Benjamini & Hochberg. The data presented in the study are deposited in the iProX repository, accession number PXD 063783.

### ELISA validation

Serum levels of FLNA and APOA4 were quantified using commercial human ELISA kits (FLNA: ELK Biotechnology, Cat# ELK4516; APOA4: ELK Biotechnology, Cat# ELK11097) according to the manufacturers’ protocols. All measured concentrations were normalized to total protein content and are expressed as ng/mL.

## Results

### Study design and patients

**FIGURE 1:**
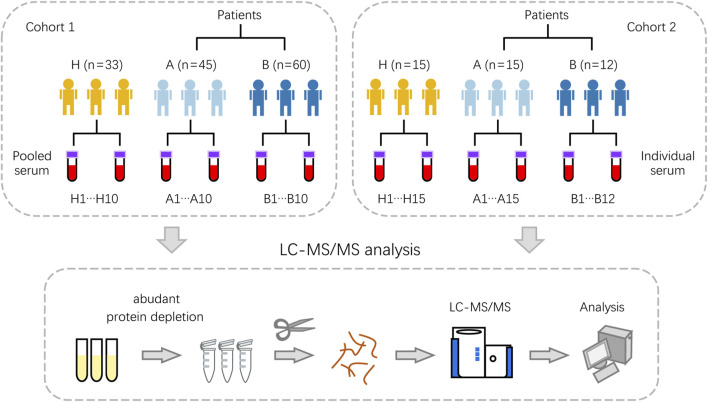
Schematic of the proteomic analysis pipeline illustrating the processing of serum samples from healthy controls, colorectal inflammatory polyp patients, and colorectal adenoma patients in both Cohort 1 and Cohort 2. Cohort 1 consisted of pooled samples from three groups: inflammatory polyps (A, n = 45), adenomas (B, n = 60), and healthy controls (H, n = 33). For each group, biological replicates were combined to generate 10 pooled samples. Cohort 2 comprised individual (non-pooled) samples from the same diagnostic categories: A (n = 15), B (n = 12), and H (n = 15). The workflow for proteomic profiling including high-abundance protein depletion, protein digestion, LC-MS/MS analysis and data processing.

For serum proteomic profiling, samples were collected from two independent cohorts (Figure 1; Supplementary Table S1). Cohort 1 comprised pooled samples from 45 patients with inflammatory colorectal polyps, 60 patients with colorectal adenomas, and 33 healthy controls. This pooling strategy was employed to ensure sufficient protein quantity for detection while mitigating inter-individual variability and maintaining biological representativeness. Cohort 2 consisted of individual samples from 15 patients with inflammatory colorectal polyps, 12 patients with colorectal adenomas, and 15 healthy controls, serving as an independent validation set. Blood contains a reservoir of potential diagnostic biomarkers. To exploit this potential, serum samples were subjected to high-abundance protein depletion, followed by reduction, alkylation, and tryptic digestion to generate peptides. Peptide samples were analyzed by nanoflow liquid chromatography-tandem mass spectrometry (LC-MS/MS) to characterize alterations in the serum proteome of colorectal adenoma patients. In two cohorts, colorectal adenoma patients exhibited a significant male predominance (75.0%), consistent with established epidemiological patterns. Additionally, no statistically significant differences were demonstrated in age or polyp number among healthy individuals, patients with inflammatory polyps, and those with adenomas (Supplementary Figure S1).

### Proteomic profiling of serum

**FIGURE 2:**
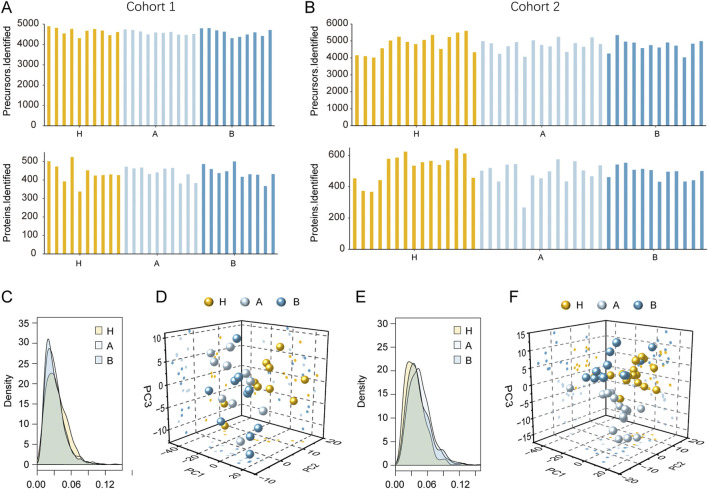
Proteomic profiling of serum from patients with colorectal polyps and H volunteers. **(A)** Distribution of identified peptides and proteins in cohort 1. **(B)** Distribution of identified peptides and proteins in cohort 2. **(C)** Coefficient of variation (CV) distribution in cohort 1. **(D)** Principal component analysis (PCA) of cohort 1. **(E)** Coefficient of variation (CV) distribution in cohort 2. **(F)** Principal component analysis (PCA) of cohort 2.

In cohort 1, a total of 5,568 peptides were identified, with per-sample counts ranging from 4,307 to 4,891. From these, 633 human proteins were quantified in at least one sample, and the per-sample protein count ranged from 337 to 524 (Figure 2A). In cohort 2, proteomic analysis identified 7,716 peptides in total (range: 4,020–5,588 per sample). Among these, 916 human proteins were quantified in at least one sample, with individual sample quantifications ranging from 267 to 524 proteins (Figure 2B). To assess the reliability of the proteomic data, we examined the raw MS/MS data and found that over 95% of peptides were matched by ≥2 spectral counts in cohort 1. The average spectral count for all peptides in this cohort was 28.9, indicating high peptide-level reliability (Supplementary Figure S2A). Furthermore, 521 proteins (82.3%) were supported by ≥2 peptides, with an average of 7.0 peptides per protein (Supplementary Figure S2B), confirming high confidence in protein-level quantification. Similarly, in cohort 2, over 95% of peptides were matched by ≥2 spectral counts, and 73.6% of proteins were supported by ≥2 peptides (Supplementary Figures S2C–D).

We further evaluated quantitative precision by calculating the coefficient of variation (CV) of protein expression across all samples. Samples H, A, and B showed high measurement precision in both cohorts, with CVs of 3.1%, 2.8%, and 2.7% in cohort 1, and 3.5%, 4.4%, and 3.9% in cohort 2, respectively (Figures 2C,E). These low CV values reflect the high reproducibility of the entire analytical workflow, including sample preparation, data acquisition, and bioinformatic processing. Principal component analysis (PCA) was performed to visualize overall proteomic profiles (Figures 2D,F). Notably, the three groups in cohort 2 showed clearer separation than those in cohort 1, suggesting improved group discrimination in cohort 2. The distribution of MS/MS spectral counts of quantified peptides and the distribution of peptide numbers of quantified proteins are shown in Supplementary Figure S2.

### Proteomic alterations associated with colorectal inflammatory polyps and adenomas

**FIGURE 3:**
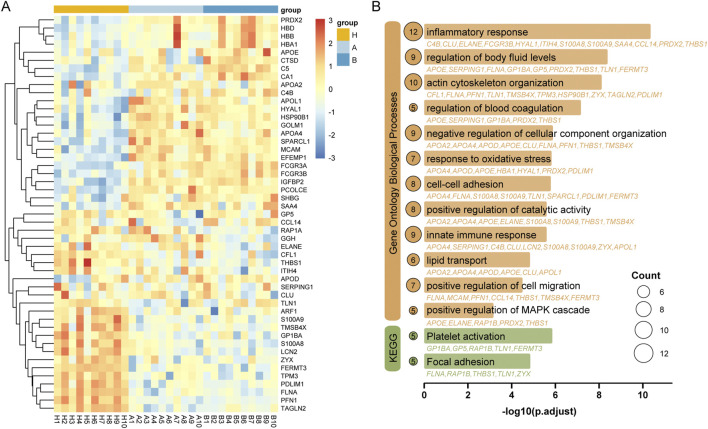
Proteomic alterations associated with colorectal inflammatory polyps and adenomas. **(A)** Heatmap visualization of statistically significant differentially expressed proteins (DEPs) with statistical significance defined as adjusted p < 0.05 in cohort 1 (one-way ANOVA with Benjamini-Hochberg correction). **(B)** Enrichment analysis of differentially expressed proteins of Gene Ontology biological process and KEGG pathway.

Serum proteomic profiling of cohort 1 revealed 49 significant DEPs (one-way ANOVA, adjusted p < 0.05) distinguishing patients with colorectal inflammatory polyps, patients with adenomas and healthy controls. Hierarchical clustering of these proteins demonstrated distinct molecular stratification, as visualized in the accompanying heatmap (Figure 3A). The DEPs were subsequently analyzed using Gene Ontology (GO) and Kyoto Encyclopedia of Genes and Genomes (KEGG) pathway enrichment analyses. Significant enrichment was observed in biological process terms and KEGG pathways including inflammatory response, fluid homeostasis regulation, actin cytoskeleton reorganization, platelet activation, and focal adhesion (Figure 3B; Supplementary Table S3).

### Biomarker exploration for differentiation of colorectal adenomas

**FIGURE 4:**
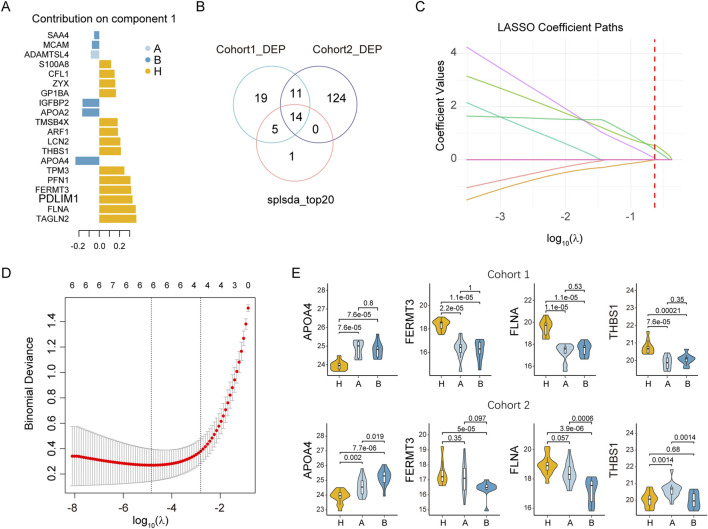
Identification of potential biomarkers for the classification of colorectal adenomas, inflammatory colorectal polyps, and healthy controls. **(A)** Sparse partial least squares discriminant analysis (sPLS-DA) of serum proteomic profiles. The analysis was performed on the three groups in cohort 1 using proteins quantified in both cohort 1 and cohort 2. The plot displays the top 20 proteins with the highest absolute contribution weights in Component 1. **(B)** Venn diagram identifying consensus proteins. The diagram compares candidate proteins identified by three independent methods: the top 20 proteins from sPLS-DA (Component 1), differentially expressed proteins (DEPs) in cohort 1 (adjusted p < 0.05), and DEPs in cohort 2 (adjusted p < 0.05). Fourteen proteins were common to all three sets. **(C)** Feature coefficient profiles in LASSO regression. The coefficient paths for the 14 initial input features are shown across the series of log(λ) values. **(D)** Parameter selection via ten-fold cross-validation. The optimal λ value was selected using the minimum criterion, as indicated by the vertical dashed line. This procedure resulted in a final model comprising four features with non-zero coefficients. **(E)** Expression of the four candidate proteins in cohorts 1 and 2. Statistical comparisons of the log2 transformed intensities between groups were performed with Welch’s t-test.

To identify potential adenoma biomarkers, we integrated the proteomic data from cohort 1 and cohort 2 using the subset of proteins quantified in both cohorts. Following batch effect removal, the overall data distribution was consistent across cohorts (Supplementary Figure S3A). Principal component analysis (PCA) further confirmed minimal inter-cohort variation (Supplementary Figure S3B), while clearly separating the three experimental groups (Supplementary Figure S3C). The top five proteins identified by sPLS-DA in cohort 1, based on variable importance in projection (VIP) scores, were transgelin-2 (TAGLN2), FLNA, PDZ and LIM domain protein 1 (PDLIM1), fermitin family homolog 3 (FERMT3), and PFN1 (Figure 4A). The intersection of 49 differentially expressed proteins (DEPs) from cohort 1, 149 DEPs from cohort 2 (one-way ANOVA, adjusted p < 0.05), and the top 20 VIP proteins from sPLS-DA analysis yielded 14 consensus candidate proteins (Figure 4B). Subsequent LASSO regression analysis of these 14 proteins identified four candidate biomarkers (Figures 4C,D). Among these, APOA4, FERMT3, and FLNA exhibited consistent expression changes in the adenoma groups of both cohorts (Figure 4E). In contrast, THBS1 was downregulated in cohort 1 but showed no significant change in cohort 2. Additionally, APOA4 and FLNA levels differed significantly between inflammatory polyps and adenomas in at least one cohort. Based on these findings, we selected APOA4 and FLNA for further validation of their serum levels.

### Validation and performance of FLNA as biomarker

**FIGURE 5:**
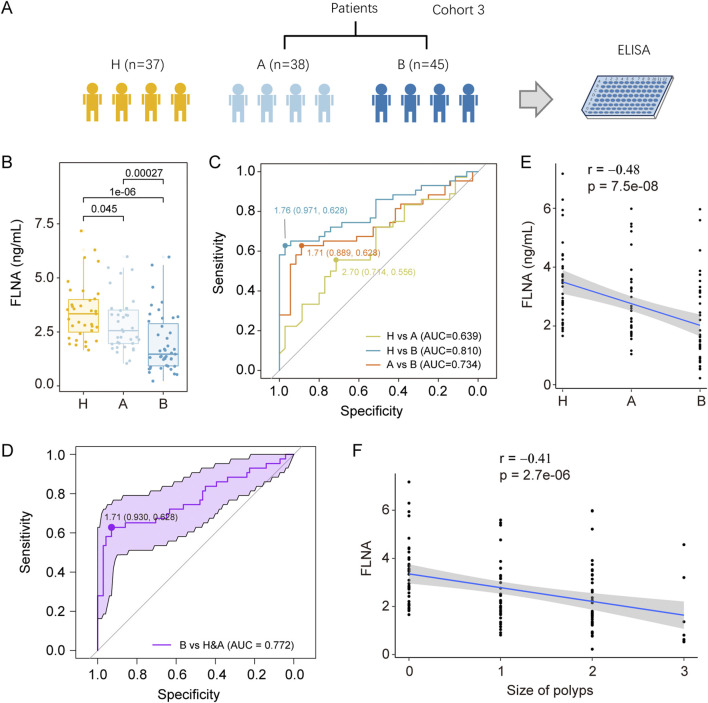
Serological validation of colorectal adenoma biomarkers. **(A)** Overview of serum sample collection from cohort 3, including H (n = 37), A (n = 38), and B (n = 45). **(B)** Serum FLNA concentrations in Cohort 3 were quantified via ELISA. Individual data points represent values from single subjects, with boxplot center lines denoting median values. To minimize outlier effects, we excluded maximum and minimum values from each group. Statistical comparisons were performed using unpaired two-sided Wilcoxon test. **(C)** The ROC curve evaluating the ability of FLNA from serum to differentiate between the three groups. The sensitivity and specificity at the optimal cutoff value are presented. **(D)** ROC curve with confidence interval of FLNA for the prediction of colorectal adenoma patients from both healthy controls and inflammatory colorectal polyp patients. Association analyses were performed to evaluate **(E)** the relationship between serum FLNA concentrations and clinical diagnosis, and **(F)** the correlation between FLNA levels and polyp size. Statistical correlations were assessed using Spearman rank correlation analysis.

To validate APOA4 and FLNA as potential biomarkers, we conducted an independent validation study in cohort 3 (Figure 5A; Supplementary Table S4), including 37 healthy controls (H), 38 inflammatory polyp patients (A), and 45 adenoma patients (B). Serum samples from these individuals were collected and then subjected to ELISA analyses to measure the serum levels of APOA4 and FLNA. Notably, the serum levels of APOA4 measured by ELISA did not correlate with the prior LC-MS/MS proteomic findings (Supplementary Figure S4). In contrast, ELISA quantification of FLNA revealed a progressive decrease in serum levels along the H-A-B pathological continuum (Figure 5B). Receiver Operating Characteristic (ROC) analysis demonstrated FLNA’s diagnostic performance with AUC values of 0.810 (95% CI: 0.713–0.907) for H vs. B discrimination, 0.734 (95% CI: 0.622–0.85) for A vs. B, and 0.639 (95% CI: 0.51–0.77) for H vs. A, indicating moderate-to-good diagnostic accuracy for adenoma detection (Figure 5C). FLNA reached an AUC value of 0.772 (95% CI: 0.673–0.871) to distinguish patients with colorectal adenoma from both healthy controls and inflammatory colorectal polyp patients (Figure 5D). At the determined cutoff, FLNA demonstrated high specificity but limited sensitivity for distinguishing B from H, B from A, and B from the combined group of H&A. The classifier showed high positive predictive values (PPV: 0.96 for H vs. B; 0.87 for A vs. B) but comparatively low negative predictive values (NPV: 0.68 for H vs. B; 0.67 for A vs. B) (Supplementary Figure S5). Serum levels of FLNA exhibited a negative correlation with the progression from normal tissue to polyp and subsequent development into adenoma (Figure 5E), as well as with the total number of polyps present (Figure 5F). Western blot analysis revealed no significant difference between inflammatory polyp patients and healthy controls, and adenoma patients showed significantly decreased FLNA expression relative to both healthy controls and inflammatory polyp cases (Supplementary Figure S6). Circulating FLNA likely derives from both passive leakage of damaged or remodeling tissues and active inflammatory secretion. The observed serum FLNA reduction may directly result from decreased tissue expression.

## Discussion

Molecular biomarkers have become indispensable tools for disease screening, diagnosis, and therapeutic monitoring in modern clinical practice. The development of robust biomarker-based diagnostic approaches holds significant promise for improving colorectal adenoma detection rates and ultimately reducing colorectal cancer incidence. In this study, we employed DIA LC-MS/MS to identify differentially expressed proteins (DEPs) in serum samples from healthy controls, patients with colorectal inflammatory polyps, and patients with colorectal adenomas. From the 49 DEPs identified in cohort 1, bioinformatics analysis revealed predominant involvement in inflammatory response, cytoskeletal reorganization, cell adhesion, and platelet activation pathways. To determine whether the DEPs identified in cohort 1 showed consistent changes in individual samples, we conducted proteomic analysis on the independent Cohort 2 and identified 25 overlapping DEPs. Notably, several significantly altered proteins, including APOA4, CLU, and SAA4, have previously been reported as potential biomarkers for colorectal cancer (Hlavca et al., 2024; Urbiola-Salvador et al., 2024; Zhu et al., 2024). Among the top 20 proteins ranked by VIP scores in the sPLS-DA model, 14 were DEPs common to both cohorts. Subsequent LASSO regression analysis identified APOA4, FERMT3, FLNA, and THBS1 as key discriminators. Based on the consistency of their expression changes in the adenoma groups across both cohorts and their differential expression between inflammatory polyps and adenomas, we selected APOA4 and FLNA for further investigation, while excluding FERMT3 and THBS1. As the ELISA results failed to confirm the serum level variations of APOA4, we therefore prioritized FLNA for subsequent analyses.

FLNA, a ubiquitously expressed cytoskeletal protein belonging to the filament protein family, serves as a critical scaffolding molecule that orchestrates cellular shape and motility through its interactions with diverse partners including integrins, transmembrane receptor complexes, adaptor proteins, and secondary messengers (Scott et al., 2006; Feng and Walsh, 2004). FLNA concurrently integrates cell structural and signaling functions and is involved in signal transduction of diverse biological processes (Stossel et al., 2001), including cell proliferation (Zhang et al., 2018), adhesion (Jain et al., 2022), migration (Stossel et al., 2001; Zhang et al., 2018; Jain et al., 2022), invasion (Xu et al., 2010), and platelet aggregation (Lopez et al., 2018). Alterations in FLNA expression levels have been observed in inflammatory conditions across multiple tissue types, including hepatitis, intestinal inflammation, nephritis, and airway inflammation (Zhang et al., 2021; Gawish et al., 2025; Lu et al., 2023; Maire et al., 2024). Our Western blot analysis of colon tissues revealed a disease status–associated decrease in FLNA expression, with significantly lower levels in adenomas compared with normal tissues. We hypothesize that tissue FLNA may enter the circulation via passive leakage or active secretion mechanisms, thereby influencing serum FLNA concentrations. However, this hypothesis requires further validation using additional clinical specimens.

Accumulating evidence indicates that FLNA dysregulation is implicated in various cancer types, including breast cancer (Xu et al., 2010), parathyroid carcinoma (Storvall et al., 2021), adrenocortical carcinoma (Esposito et al., 2025), and prostate cancer (Di Donato et al., 2021). Furthermore, FLNA has been reported to be significantly downregulated at the transcriptional level in human colorectal adenoma tissues (Zhang and Fu, 2025), with aberrant expression also observed in colorectal tumor tissues. The expression pattern and functional mechanisms of FLNA in colorectal cancer (CRC) remain subjects of ongoing debate. While some studies report FLNA downregulation in CRC tissues, others document significant upregulation. Notably, evidence from clinical studies indicates that reduced FLNA expression correlates with poorer overall survival in CRC patients. Mechanistically, calpain-1-mediated FLNA proteolysis contributes to FLNA downregulation, which is associated with adverse clinical outcomes (Xu et al., 2019). Consistently, low FLNA expression has been identified as a risk factor for unfavorable prognosis (Wang et al., 2022). Functional studies further demonstrate that FLNA silencing in colorectal cancer HT29 cells attenuates Snail-mediated cell adhesion and promotes cell migration (Wieczorek et al., 2017). Additionally, WTAP upregulation in colon cancer downregulates FLNA expression through m6A modification at the 3'UTR region (Huang et al., 2023). More recently, FLNA has been identified as a key mediator of disulfidptosis in CRC, where its knockdown suppresses tumor cell migration and invasion (Li et al., 2025). In line with a potential tumor-promoting role, elevated FLNA protein levels have been observed in colon cancer tissues compared to adjacent non-cancerous counterparts. Correspondingly, *in vitro* experiments confirm that FLNA silencing significantly impairs cellular migration and proliferation capacity (Liu et al., 2025). To our knowledge, no studies have yet reported whether blood levels of FLNA differ among healthy individuals, inflammatory polyp patients, and adenoma patients. Furthermore, no biomarker currently exists that can simultaneously distinguish healthy individuals from adenoma patients and differentiate non-neoplastic polyps from adenomas. Our data identify FLNA as a novel biomarker for colorectal adenoma. ROC analysis demonstrated FLNA’s ability to discriminate both between healthy controls and adenomas, and between inflammatory polyps and adenomas. When healthy individuals and inflammatory polyp patients were combined into a single group, FLNA still effectively distinguished adenomas from this combined group. FLNA exhibited high specificity, indicating strong recognition capability for adenomas, though its somewhat lower sensitivity reflects variability in identifying true positive cases. These findings suggest FLNA’s potential clinical utility in guiding colonoscopy referrals for symptomatic patients.

Due to limitations in hospital patient admissions, we only collected and analyzed inflammatory polyps among non-neoplastic polyps, excluding other subtypes. Our comparative analysis of pooled and individual serum samples revealed that while pooling reduces intra-group variability and minimizes individual-specific noise in differential protein screening, it may also obscure inter-group distinctions and be influenced by extreme values. Moreover, sample pooling precludes clinical correlation analysis. In contrast, analysis of individual samples enables clinical association studies, fully captures individual variations, and mitigates outlier effects. When funding and instrument availability permit, individual sample analysis is the preferred approach. Additionally, the adoption of advanced methods such as nanomagnetic bead-based depletion of high-abundance proteins could facilitate the quantification of serum proteins across a wider dynamic range, potentially yielding further discoveries. Although our current findings are promising, we acknowledge several study limitations, including the restricted sample size and single-center recruitment. Future work should involve expanded cohorts, improved data quality, and multi-center validation studies to enhance the generalizability of the results.

## Data Availability

The data presented in this study are deposited in the iProX repository, accession number: PXD063783.
