# Supplementary material for: Quantitative proteomic analysis reveals potential serum diagnostic markers for colorectal adenoma
Source: Front Mol Biosci. 2025 Nov 24;12:1628587. doi: 10.3389/fmolb.2025.1628587 (PMC12682900; doi:10.3389/fmolb.2025.1628587)
Supplement: Supplementary file 7 [file DataSheet1.docx]

**Supplemental Information**

**Supplementary figures for Quantitative Proteomic Analysis Reveals Potential serum diagnostic markers for colorectal adenoma**

This file includes:

Figure S1 to S6

Figure S1 Demoghraphics and clinical characteristics (including sex, age, and polyp number) of the discovery and test cohorts.

Figure S2 The distribution of MS/MS spectral counts of quantified peptides, and peptide numbers of quantified proteins in discovery phase and test phase.

Figure S3 Batch effect correction on proteins common to cohort1 and cohort 2.

Figure S4 ELISA quantification results of APOA4 in serum.

Figure S5 Confusion matrix related to Figure 4.

Figure S6 FLNA expression levels in clinical colon tissues.


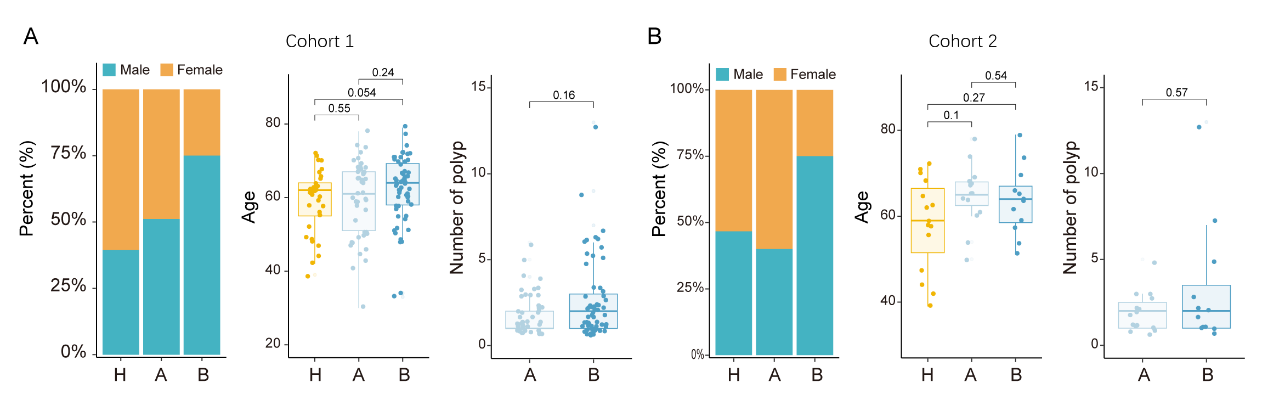
Figure S1


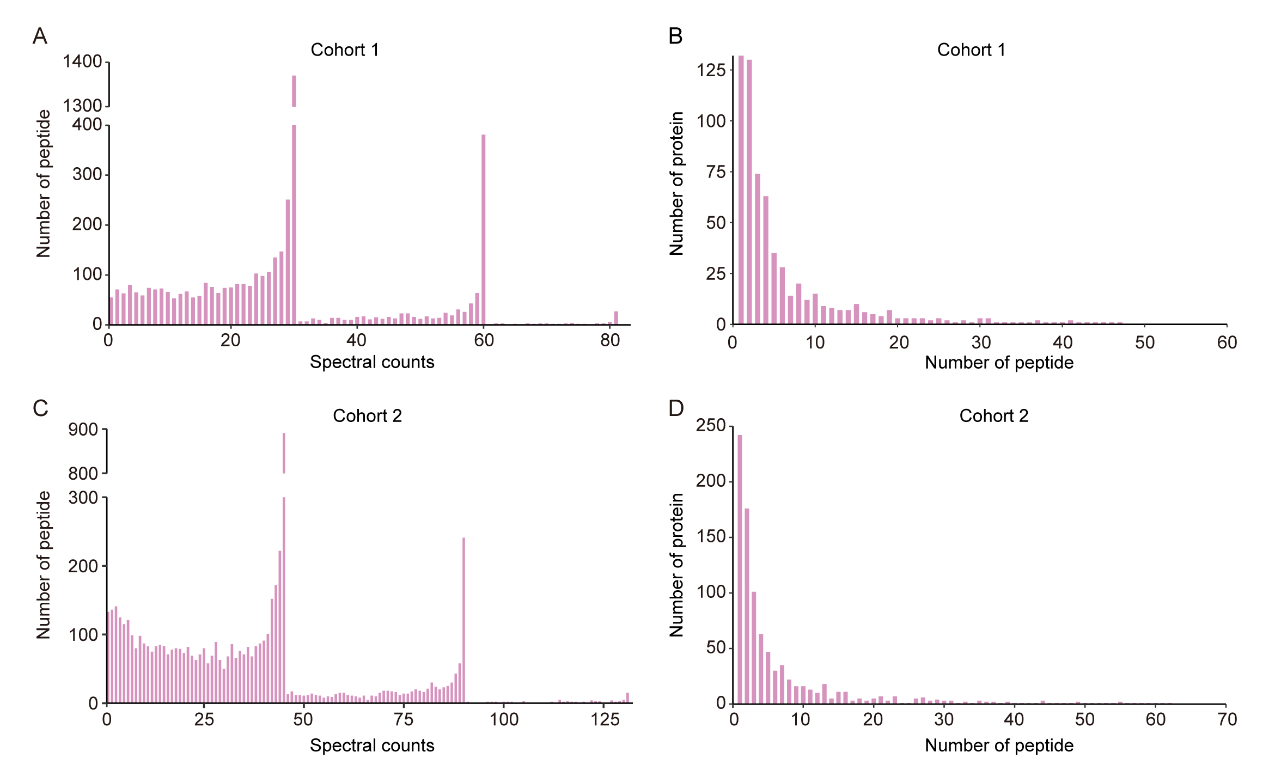
Figure S2


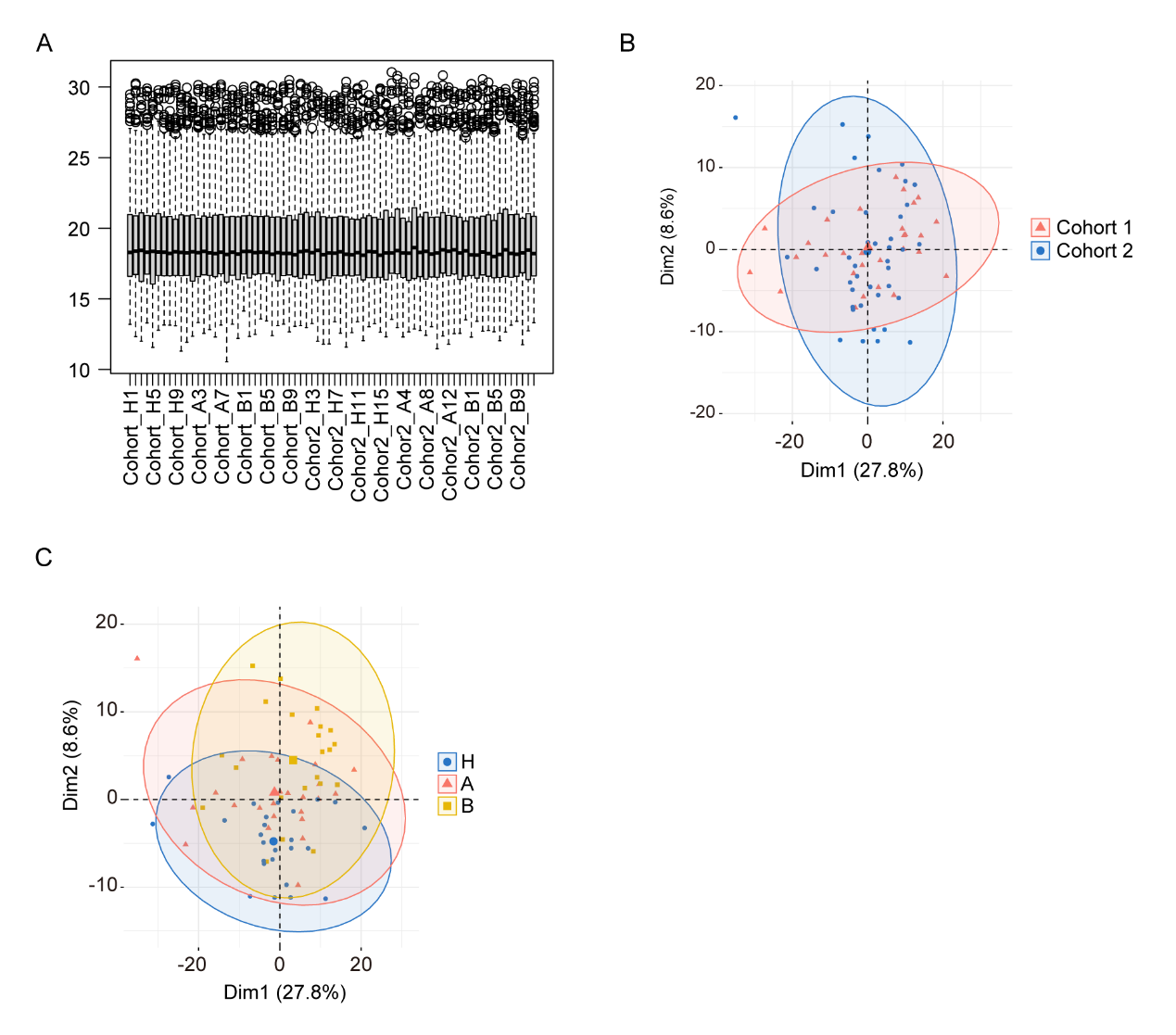
Figure S3

Figure S4
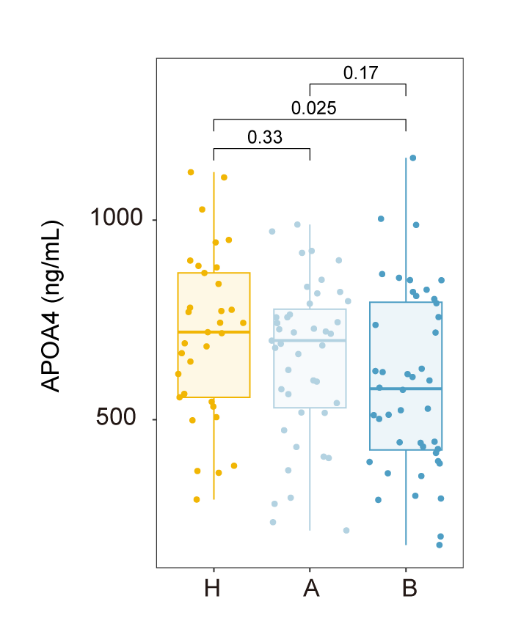


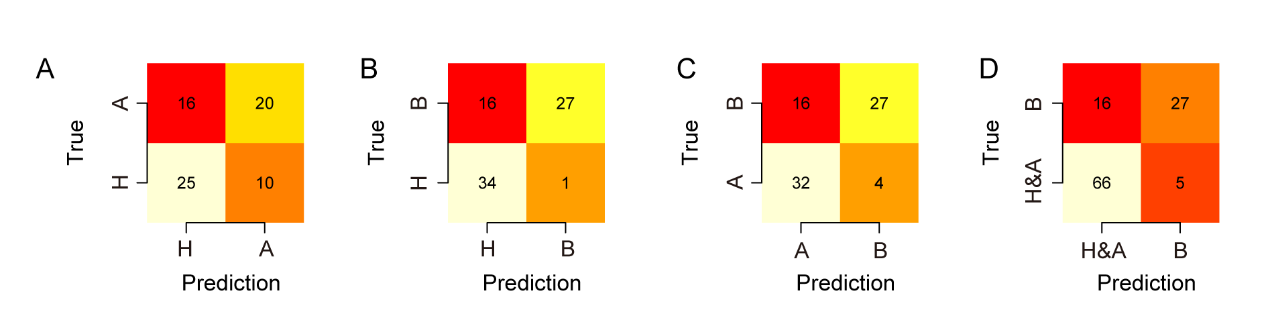
Figure S5


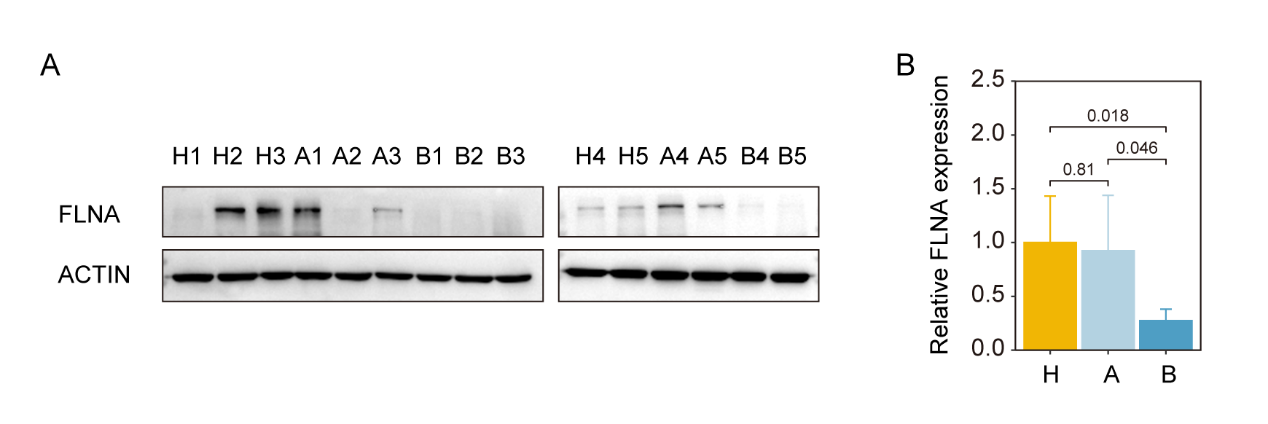
Figure S6
